# Effect of Alpha-Lipoic Acid on the Development, Oxidative Stress, and Cryotolerance of Bovine Embryos Produced In Vitro

**DOI:** 10.3390/vetsci12020120

**Published:** 2025-02-02

**Authors:** Mariana Moreira dos Anjos, Gabriela Rodrigues de Paula, Deborah Nakayama Yokomizo, Camila Bortoliero Costa, Mariana Marques Bertozzi, Waldiceu Aparecido Verri, Amauri Alcindo Alfieri, Fábio Morotti, Marcelo Marcondes Seneda

**Affiliations:** 1 Laboratory of Animal Reproduction, University of Londrina (UEL), Londrina 86057-970, PR, Brazil; mariana.moreira.anjos@uel.br (M.M.d.A.); gabrielarodrigues.p@uel.br (G.R.d.P.); deborah.yokomizo@uel.br (D.N.Y.); camilabcg@uel.br (C.B.C.); fabiomorotti@uel.br (F.M.); 2 Laboratory of Pain, Inflammation, Neuropathy, and Cancer, Department of Immunology, Parasitology, and General Pathology, Center of Biological Sciences, Londrina State University, Londrina 86057-970, PR, Brazil; marianambertozzi@gmail.com (M.M.B.);; 3 Laboratory of Animal Virology, University of Londrina (UEL), Londrina 86057-970, PR, Brazil

**Keywords:** antioxidants, cryopreservation, embryo quality, reactive oxygen species

## Abstract

Oxidative stress (OS) induced by an imbalance in reactive oxygen species (ROS) levels in vitro impairs embryonic development. Here, we assessed the effects of alpha-lipoic acid (ALA) in in vitro production media on OS reduction, embryonic development, and cryotolerance of bovine embryos. We evaluated the effects of adding different concentrations of ALA (2.5, 5, 10, and 25 μM) to in vitro maturation (IVM) or in vitro culture (IVC) medium on embryonic development. We also determined the effects of adding ALA (25 μM) to the IVM and IVC medium in the same routine on the development and quality of embryos, ROS levels, and cryotolerance. Embryos were produced in vitro using conventional protocols for each treatment. The inclusion of ALA in the IVM and IVC media did not affect the development or quality of embryos; however, it reduced ROS levels in grade II embryos and increased hatching after 12 h on day 7 in grade I embryos and on day 8 in grade II embryos after warming. These findings prompt questions regarding the potential of ALA in improving embryo metabolism, considering the initial embryo recovery in the first few hours of embryo warming.

## Simple Summary

Antioxidants are used to control reactive oxygen species for optimizing in vitro embryo development. We evaluated the effects of including alpha-lipoic acid (ALA) in in vitro production media on the alleviation of oxidative stress, development, and cryotolerance of bovine embryos. Embryos were produced in vitro using conventional protocols, with the inclusion of ALA in production media at different stages. Embryo development and hatching kinetics were not affected when different ALA concentrations were included only in the maturation or culture medium or in both. However, ALA inclusion in both the media reduced reactive oxygen species levels in grade II embryos and increased hatching after 12 h on day 7 in grade I embryos and on day 8 in grade II embryos after warming. These findings prompt questions regarding the potential of ALA in improving embryonic metabolism.

## 1. Introduction

The significant increase in the production of bovine embryos in recent decades [1] has led to the development of new protocols and optimization of parameters to enhance the production of embryos and improve their quality. The environment in which embryos are produced in vitro causes structural, cellular, and metabolic changes with respect to in vivo-produced embryos [2]. Oxidative stress (OS), the result of imbalanced generation and elimination of reactive oxygen species (ROS), is one of the factors responsible for the differences between embryos produced in different systems [3].

ROS acts as a second messenger in cell signaling and control pathways [4,5,6,7,8]. However, at supraphysiological levels, ROS damages cells via different mechanisms, including lipid peroxidation [9], cell membrane damage [10], protein aggregation and degradation [11], and DNA damage [12]. These factors contribute to the differences in the competence of in vitro-produced embryos [2]. Increased ROS levels have been observed in the in vitro production (IVP) of embryos from several species, including cattle [13], mice [14], and humans [15], and are associated with negative effects on the development, quality, and viability of embryos [16]. Furthermore, the cryotolerance of these embryos is impaired by high ROS and lipid concentrations, which increase lipid peroxidation [17]. Supplementation of production media with antioxidant compounds has been explored as a method for minimizing the damage induced by excessive ROS and OS in the in vitro environment [2].

Alpha-lipoic acid (ALA) is an antioxidant that inactivates free radicals and ROS [18,19]. It plays a role in the repair of other antioxidants, such as vitamins C and E [20,21,22], and modulates intracellular glutathione and ascorbate levels [23,24]. In addition, ALA is involved in mitochondrial energy production [25], and glucose [26,27] and lipid [28] metabolism. Considering the varied biochemical characteristics of ALA [29], its application in tissue [30] and embryo culture [31] has been explored. Recent studies have revealed the effectiveness of ALA in maintaining follicular integrity in equine [30] and bovine preantral follicle cultures [32]. ALA also improves the antioxidant capacity of oocytes [33,34], sperm [31,35], and embryos [36,37]. In addition, it attenuates the toxic effects of ethanol in ovine oocytes [38], oxidative damage by lipopolysaccharides in mouse embryos [39], and thermal stress in porcine parthenotes [40].

The effect of ROS in the in vitro environment is complex—free radicals can exert effects ranging from cell deterioration to cell promotion, depending on the rate of free radicals and stage of embryonic development [41]. Corroborating this complexity, previous studies have indicated that high concentrations of ALA could adversely affect the development of these structures [34,39,40,42]. Although some studies have shown the antioxidant effects of ALA in vitro, the factors to be considered when including ALA in IVP of bovine embryos, such as the concentration and stage of inclusion and its influence on the quality and cryotolerance of embryos, have not yet been fully elucidated. It is believed that supplementing media with ALA at different stages of IVP minimizes the negative effects of OS, favors proper development, and improves the cryotolerance of bovine embryos. Therefore, we explored the potential of including ALA in the IVP media in reducing OS and consequently ROS levels, with the aim of improving the cryosurvival of bovine embryos.

## 2. Materials and Methods

All procedures were performed in accordance with the guidelines of the University Committee for Ethics in Animal Research (CEUA; protocol no. 031.2024) of the State University of Londrina.

### 2.1. Production Media and Reagents

All in vitro embryo production media and solutions used for vitrification and warming were obtained from ABS Global Brazil^®^ (Mogi Mirim, São Paulo, Brazil) ). Alpha-lipoic acid (T1395, CAS 1077-28-7) was obtained from Sigma-Aldrich^®^ (St. Louis, MO, USA), and 2′,7′-dichlorodihydrofluorescein diacetate probe (H2DCFDA, CAS D399) was purchased from Invitrogen^®^.

### 2.2. Collection, Transportation, and Processing of Ovaries

Female bovine ovaries were obtained from a local slaughterhouse, and cumulus-oocyte complexes (COCs) were recovered by aspiration of the antral follicles (2–8 mm). COCs surrounded by a minimum of three layers of cumulus cells and homogeneous cytoplasm [43] were selected for in vitro maturation (IVM).

### 2.3. In Vitro Production of Bovine Embryos

The selected COCs were placed in drops containing 100 µL of TCM-199 maturation medium supplemented with 10% (*v/v*) fetal bovine serum, 5 mg luteinizing hormone, 0.5 mg follicle-stimulating hormone, 1 mg estradiol, 2.2 mg pyruvate, and 50 mg gentamicin/mL. The cells were then subjected to IVM for 22–26 h.

After maturation, the COCs were washed in Hepes-buffered TCM-199 medium and placed in 100 µL drops of fertilization medium, composed of Tris-buffered medium (TBM) supplemented with 8 mg/mL fatty acid-free bovine serum albumin (BSA) and 1 mM glutamine. Semen from a single bull was used for fertilization after selection using a Percoll gradient, with the final sperm concentration adjusted to 1 × 10^6^ live sperm/mL. Between 18 and 20 h after fertilization, probable zygotes were denuded by continuous pipetting, washed in Hepes-buffered TCM-199 medium, and cultured in vitro in synthetic oviduct fluid (SOF) medium supplemented with 8 mg/mL of fatty acid-free BSA. On the third day of culture, the first feeding with SOF medium was performed, and on the fifth day of culture, the second feeding with glucose SOF medium was performed.

All steps were carried out in a controlled environment at 38.5 °C, in an atmosphere of 5% CO_2_ in air and saturated humidity. The IVM medium and SOF were supplemented with ALA according to the treatments.

### 2.4. Experimental Design

**Figure 1:**
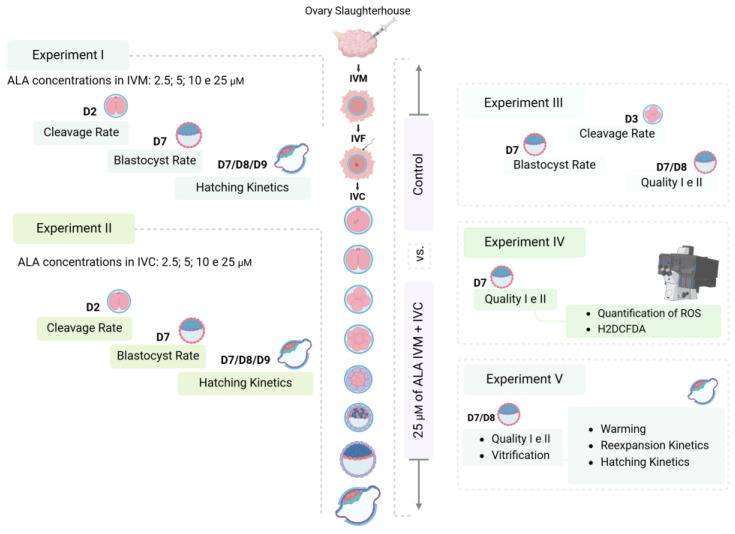
Schematic of the experimental design.

ALA was dissolved in ethanol and ultrapure water to prepare a stock solution. This stock solution was diluted with the production medium to obtain solutions with the required treatment concentrations. All experiments included a control group (without ALA). The first series of experiments was aimed at evaluating the effect of 0, 2.5, 5, 10, and 25 µM of ALA on the cleavage rate (cleaved structures/possible zygotes), blastocyst rate (blastocysts/possible zygotes), and hatching kinetics (hatched/blastocysts) in both the IVM medium (Figure 1; Experiment I) and in vitro culture medium (Experiment II). The other experiments were aimed at evaluating the effects of supplementing the IVM and IVC media in the same routine with control or 25 μM ALA on the development and quality of embryos (Experiment III), levels of ROS (Experiment IV), and cryotolerance of embryos (Experiment V).

#### 2.4.1. Experiment I and II: Evaluation of the Effects of Including Different Concentrations of ALA in the Production Medium on Embryonic Development

The different concentrations were tested separately at each production stage. In experiment I, the IVM medium was supplemented with ALA at 0, 2.5, 5, 10, and 25 µM (*N* = 2935, 8 replicates). In experiment II, the SOF medium was supplemented with ALA at 0, 2.5, 5, 10, and 25 µM (*N* = 2201, 8 replicates). The cleavage and blastocyst rates were assessed on days 2 and 7 of culture, respectively. Hatching kinetics were also evaluated on days 7, 8, and 9 of culture.

#### 2.4.2. Experiment III: Effects of Including ALA in the Maturation and Culture Medium on the Development and Quality of Embryos

The IVM and SOF medium, from the same routine, were supplemented with ALA at 0 and 25 µM (*N* = 3036, 9 replicates). Embryos were produced as described previously. The cleavage rate, blastocyst rate, and hatching kinetics were evaluated on days 3, 7, 8, and 9. On days seven and eight of culture, expanded embryos were assessed for grade I and II quality [44].

#### 2.4.3. Experiment IV: Measurement of Intracellular ROS Levels Using the Dichlorofluorescein Assay

To evaluate the effect of ALA on OS, embryo production was carried out as described above, but with the inclusion of 25 µM ALA in both the maturation and culture media of the same production routine.

The intracellular content of ROS was quantified using the fluorescent probe H2DCFDA, as described by Bain et al. [45], with adaptations. Briefly, pool quality grade I and II expanded blastocysts from the control (*N* = 38) and ALA (*N* = 42) groups were washed and incubated for 20 min in phosphate-buffered saline (PBS) with 0.01% PVA and 5 µM H2DCFDA. Subsequently, they were washed in three drops of PBS with 0.01% PVA and immediately photographed using a confocal microscope. The settings were identical (a total of 4 replicates; Leica TCS SP8; excitation 495 nm and emission 520 nm) for all evaluated structures. To quantify the intensity of the emitted fluorescence, the images were analyzed using the ImageJ software (ImageJ version 1.54g, National Institutes of Health, Bethesda, MD, USA). The total area of each blastocyst was measured using a freehand tool to delimit the cytoplasm of each blastocyst and measured in pixels (ImageJ software). The background signal intensity was subtracted from the measured values. The relative fluorescence intensity of each blastocyst was obtained by dividing the total fluorescence by the total area.

#### 2.4.4. Experiment V: Evaluation of Cryotolerance Through Vitrification

To assess the impact of ALA on cryotolerance, the inclusion of 25 µM ALA in the IVM and IVC medium from the same production routine was also considered. Expanded blastocysts (*N* = 325, 9 replicates) on days 7 and 8 were assessed for quality. Grade I and II embryos were selected according to the International Embryo Transfer Society guidelines [45], subjected to vitrification using the open-pulled straw (OPS) technique developed by Vajta et al. [46], and stored in liquid nitrogen until warming.

During warming, the OPSs were removed from liquid nitrogen, and the tip of each OPS was dipped into the well of a four-well plate containing maintenance solution. Blastocysts were washed and transferred to culture plates. Re-expansion kinetics were assessed at 0, 12, and 24 h after warming, and the hatching kinetics at 12, 24, 48, and 72 h were evaluated.

### 2.5. Statistical Analysis

The effects of ALA addition on IVM and IVC were analyzed using ANOVA and a generalized linear model. Treatment (control and ALA concentrations) was considered a fixed factor, the in vitro fertilization (IVF) routine was a random factor, and the number of COCs was a covariate. In the presence of a significant effect, Tukey’s post hoc test was used. For the analysis of ROS, the effects of treatment (control and ALA) and embryo quality (GI and GII) were considered fixed factors, and the IVF routine was considered a random factor. The treatment × quality interaction effects were also considered. The relative fluorescence intensity data for ROS detection were compared using the Tukey’s test as a post-hoc mean test. For cryotolerance variables, the data were previously analyzed for normal distribution using the Shapiro–Wilk test and for homogeneity of variances using the *F*-test. Data with normal distribution and homogeneous variances were analyzed using *t*-tests. Nonparametric data were analyzed using the Mann–Whitney *U* test. For descriptive analysis, data were presented as mean (M) and standard error (SE) of the mean. All statistical analyses were carried out using the Minitab^®^ statistical program, version 24.2.1. The significance level to reject H0 (the null hypothesis) was 5%; therefore, a significance level ≤ 0.05 was considered to indicate the effect of categorical variables and their interactions.

## 3. Results

### 3.1. Effects of Supplementing the Production Medium with Different ALA Concentrations on Embryonic Development

No differences were observed in the cleavage and blastocyst rate between the control and 2.5, 5, 10, and 25 µM ALA treatment groups (*p* > 0.05; Table 1). Similarly, no differences were found in hatching on days 7, 8, and 9 or in the total hatching rate between the control and different ALA groups. Therefore, the inclusion of ALA in the IVM medium at the evaluated concentrations did not interfere with embryonic development (*p* > 0.05; Table 1).

No differences were observed in the cleavage, blastocyst rate, and hatching between the control and different ALA treatment groups (*p* > 0.05; Table 2). Therefore, the inclusion of ALA in the IVC medium at the evaluated concentrations did not interfere with embryonic development.

### 3.2. Effect of Including ALA in the Maturation and Culture Media on the Development and Quality of Embryos

With regard to the development and quality of embryos, no differences were observed in cleavage and blastocyst rates between the control and ALA treatment groups. Similarly, no differences were noted in the number of grade I and II embryos on days 7 and 8 and in the number of vitrified embryos between the control and ALA groups (*p* > 0.05; Table 3).

### 3.3. Measurement of Intracellular ROS Levels Using Dichlorofluorescein Assay

**Figure 2:**
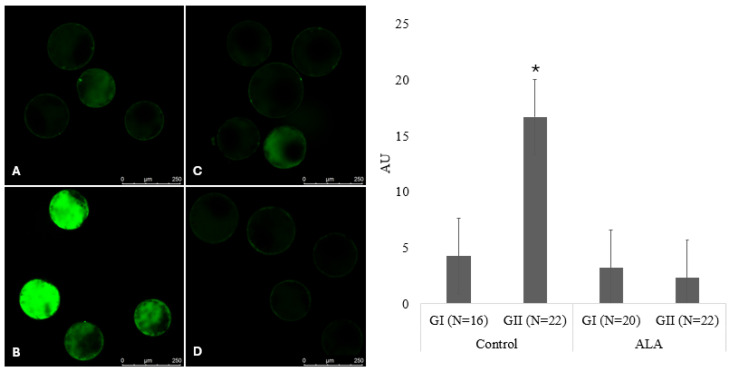
Fluorescence intensity generated by the DCF probe in the groups of embryos produced in MIV and CIV media with or without alpha-lipoic acid (ALA) supplementation. (**A**) grade I embryos from the control group (4.25 ± 1.28); (**B**) grade II embryos from the control group (16.65 ± 3.95); (**C**) grade I embryos from the ALA group (3.19 ± 1.34); (**D**) grade II embryos from the ALA group (2.32 ± 0.33). The fluorescence intensities are depicted in arbitrary units (AU). * Above each bar represent significant differences (*p* ≤ 0.05).

The average fluorescence intensity per unit area, determined by detecting the DCF probe, in fresh expanded grade I and II blastocysts from the control groups was 4.3 ± 1.3 and 16.7 ± 3.9, respectively, and that of grade I and II blastocysts from the ALA group was 3.2 ± 1.3 and 2.3 ± 0.3, respectively. The overall mean relative fluorescence intensity was influenced by the treatment (*p* < 0.0001), the quality of the embryo (*p* = 0.01), and the interaction between the factors (*p* = 0.008), with the highest mean relative fluorescence observed for control grade II embryos (Figure 2). This indicates that ALA tends to reduce ROS levels, especially in structures with lower-quality grades, which may positively affect their metabolism.

### 3.4. Evaluation of Cryotolerability Through Vitrification

The expansion rate at 0, 12, and 24 h and the total number of grade I embryos on day 7 after warming were not affected by ALA treatment (*p* > 0.05; Table 4). The hatching rate at 24, 48, and 72 h and the total number of day 7 grade I embryos after warming also did not differ; however, hatching at 12 h was higher (*p* = 0.047) for day 7 grade I embryos produced in medium supplemented with ALA (37.3%) than that in the control (12.2%). ALA had a positive effect on initial embryo recovery in the first few hours after warming.

The expansion rate (0, 12, and 24 h and total) and hatching (12, 24, 48, and 72 h and total) of day 7 grade II embryos after warming were not affected (*p* > 0.05) by ALA treatment. Similarly, ALA supplementation did not affect (*p* > 0.05) the expansion rate (0, 12, and 24 h and total) or hatching (12, 24, 48, and 72 h and total) of grade I embryos on day 8 after warming (Table 5). The expansion rate (0, 12, and 24 h and total embryos) and hatching (24, 48, and 72 h and total) on day 8 in grade II embryos after warming were not affected (*p* > 0.05) by ALA treatment; however, hatching at 12 h was higher (*p* = 0.048) in the ALA-supplemented group. Additionally, a trend (*p* = 0.077) for increased total hatching was observed on day 8 in grade II embryos supplemented with ALA after warming.

## 4. Discussion

One of the current challenges in IVP of embryos is improving embryo quality and developing protocols to optimize post-cryopreservation success. In this study, we evaluated the potential effects of ALA at different stages of IVP on the reduction in OS and the quality and developmental capacity of bovine embryos before and after cryopreservation. The harmful effects of cumulative stress have been shown to be partially mitigated by the addition of antioxidants to culture media [47,48,49].

The in vitro conditions to which gametes are exposed contribute to increased ROS production, which affects the morphology and functionality of blastomeres and even changes the extent of gene transcription and epigenetic disorders [50,51]. As maturation is one of the main processes in the production of embryos, we evaluated the effects of including different concentrations of ALA in the IVM medium on bovine embryo development. Although the balance between ROS production and cellular antioxidant defense mechanisms is essential during IVM [52,53,54], the inclusion of different concentrations of ALA in the IVM medium did not affect the cleavage rate, blastocyst rate, or hatching kinetics.

A similar effect was observed by Zhang et al. [33]. They reported that supplementing the medium with 25 μM ALA during IVM of goat oocytes after somatic cell nuclear transfer had no effect on cleavage. However, they also reported increased oocyte maturation, reconstruction of embryos, blastocyst formation, and hatching [33]. Positive effects of other concentrations of ALA in IVM on oocyte maturation and bovine embryo development (5 μM and 10 μM) [34], on the maturation, early cleavage, and development of goat parthenogenetic blastocysts (25 μmol/L of ALA) [36], and on the growth and development of secondary preantral follicles in vitro [30,55] have been reported. The positive effects of including ALA in the maturation medium are possibly associated with the fact that, during IVM, oocytes acquire an intrinsic capacity to develop until the embryonic genome is activated after fertilization [56,57].

In many species, proteins and RNAs are stored in the cytoplasm during oocyte maturation and regulate early embryonic development [58]. The processes involved in the first cleavage of the embryo depend on conditions during oocyte maturation [59]. Therefore, the potential of ALA in improving the competence for embryonic development possibly occurs via mediating the maintenance of the total antioxidant capacity of oocytes during the culture period [36] by reducing ROS levels and positively modulating the expression of genes related to antioxidant potential and cell apoptosis [33,36,38]. This also justifies the potential of ALA (25 µM) in attenuating the toxic effect of ethanol (1%) on the oocyte and on the development of sheep blastocysts [38].

These developmental events are affected by external factors and changes in the culture system, causing alterations in the transcription pattern [60,61,62]. Conditions that lead to a reduction in OS and a balance in ROS levels have shown promise for increasing the embryo production rates [37,63,64]. However, when we added different concentrations of ALA to the IVC medium, we found no effect on the cleavage rate, blastocyst rate, and hatching kinetics of bovine embryos.

Previous work is in contradiction with our findings, as ALA supplementation in the IVP medium was reported to promote improvements in embryo development, reduce OS, and increase the viability of mouse embryos [47,49]. Furthermore, low concentrations (2.5 µM) of ALA in the first 24 days of IVC increased the number of blastocysts and hatching, as well as the total number of bovine blastocyst cells [37]. This may be associated with ALA being soluble in lipids and water, easily crossing different organelles such as mitochondria, and participating in the elimination of free radicals and in the repair and reconstruction of other antioxidants [20,21,22].

Although previous studies have analyzed the supplementation of ALA in the IVM and IVC media separately, to the best of our knowledge, there has been no evaluation of the inclusion of this antioxidant in both stages of bovine embryo production. We hypothesized that the inclusion of 25 µM ALA in the IVM and IVC stages would reduce OS and improve the quality, development, and cryotolerance of bovine embryos. During IVM, oocytes acquire the intrinsic capacity to develop until the embryonic genome is activated [56,57]. Between the 2 and 4 cell stages, in vitro embryos show a peak in ROS production [65], and smaller amounts of antioxidants are generated in this window [66]. The “developmental block,” which occurs at the 8-cell stage [67], indicates that embryonic transcription is remarkably responsive to culture conditions [68].

In the early stages of embryo development, the pentose phosphate pathway produces reduced glutathione (GSH), which protects against peroxidation, and nucleotide precursors [69]. ALA plays a role in the intracellular recycling of GSH [70]. Therefore, ALA supplementation during IVP may improve embryonic development. Although the presence of ALA in the IVM and IVC media did not affect embryo development or the number of grade I and II embryos on days 7 and 8, a reduction in ROS levels was observed in grade II embryos. It is important to emphasize that ROS can damage DNA, causing breaks and mutagenic changes [71], interfering with the expression of transcription factors and molecular control of blastomeres [16,62].

The inclusion of antioxidants has shown promise in improving in vitro embryo development via the regulation of intracellular and extracellular microenvironments by reducing ROS toxicity [49]. However, it is assumed that the effect of ALA supplementation in embryo IVC medium depends on the concentration used [39,40,42], which may be associated with its pro-oxidant effects [72], and may also be affected by the composition of the medium, such as the presence or absence of amino acids and fetal bovine serum [42].

ROS, generated by external sources and energy metabolism pathways, such as mitochondrial phosphorylation and glycolysis, are the main promoters of OS [73,74]. We observed that the inclusion of 25 µM ALA in the IVM and IVC media together promoted a reduction in the average relative fluorescence of ROS in grade II embryos. ALA has been reported to protect mouse embryos against OS by stimulating the expression of antioxidant genes [75,76], increasing the number of bovine embryo cells [34,36], and increasing the number of grade I embryos on day 7 [37].

Embryo selection based on morphological qualities is a prerequisite for successful cryopreservation [77]. However, the viability of in vitro and in vivo origin embryos after freezing and thawing cycles varies [78,79]. Cell quality can be impaired by the increased production of free radicals, which stimulate embryonic death through lipid peroxidation [80], and the percentage of cells that undergo apoptosis in the vitrification process influences the ability of the embryo to resume development [81].

Some strategies are used in the culture system to increase embryo quality and cryotolerance, which are affected by suboptimal in vitro culture conditions [82]. Although we did not observe any difference in the number of vitrifiable embryos, we made the following salient observations with the inclusion of ALA: a reduction in ROS in grade II embryos; an increase in hatching in grade I day 7 embryos after 12 h; an increase in hatching in grade II day 8 embryos after 12 h; and a tendency for total hatching for embryos from day 8 to grade II. These data raise possible questions regarding the potential of ALA to improve embryo metabolism, considering the initial embryo recovery in the first few hours after embryo warming, further emphasizing its effect on day 8 grade II embryos.

Valente et al. [81] compared the speed and ability of bovine embryos to resume development after cryopreservation and classified them into cryoresistant and noncryoresistant embryos. They reported that cryoresistant embryos showed an increased total number of cells and a reduced apoptotic index; embryos with a lower apoptotic percentage were able to re-expand the blastocele, whereas a higher number of cells was found in embryos that hatched at 24 and 48 h; embryos that resumed development and hatching at 24 and 48 h after warming showed a higher number of cells. These findings confirm that the total number of cells and apoptotic index are directly correlated with the speed and ability to resume development after cryopreservation [81].

Survival rates after cryopreservation are important indicators of the quality and viability of embryos during in vitro evaluations. Although the survival rates in this study were not affected by the inclusion of ALA, the hatching rate at 12 h suggested a possible positive influence on embryo metabolism. Some studies have reported a reduction in the apoptotic index [33], an increase in the total number of cells [34,36], and the expression of genes related to antioxidant capacity (GPX4 and SOD1) [36,38] and apoptosis [33].

## 5. Conclusions

ALA supplementation in in vitro embryo production media (IVM and/or IVC) did not affect embryo development, hatching kinetics, and cryotolerability after vitrification. However, at 25 µM, ALA (IVM and IVC) reduced ROS levels in grade II embryos and increased hatching of day 7 grade I embryos at 12 h and of day 8 grade II embryos after warming.

## Figures and Tables

**Table 1:** Cleavage rates, blastocyst rates, and hatching kinetics of cumulus-oocyte complexes (COCs) subjected to in vitro maturation in the presence of different alpha-lipoic acid (ALA) concentrations.

Group	COCs	Cleavage	Blastocyst	Hatching Day 7	Hatching Day 8	Hatching Day 9	Total Hatching
Control	609	441 (74.4 ± 4.8)	182 (32.3 ± 5.8)	13 (7.2 ± 3.8)	58 (32.4 ± 6.4)	35 (19.2 ± 2.8)	106 (58.8 ± 8.7)
2.5 µM ALA	560	407 (72.6 ± 4.8)	174 (32.1 ± 4.9)	19 (10.7 ± 3.9)	60 (34.2 ± 5.9)	36 (18.2 ± 4.4)	115 (63.1 ± 7.9)
5 µM ALA	583	440 (74.8 ± 6.0)	177 (31.9 ± 6.3)	10 (4.3 ± 2.0)	60 (27.3 ± 6.4)	37 (23.1 ± 6.8)	107 (54.7 ± 9.3)
10 µM ALA	583	419 (72.7 ± 4.5)	197 (36.1 ± 5.4)	13 (6.3 ± 2.0)	69 (31.9 ± 6.4)	31 (17.0 ± 4.6)	113 (55.2 ± 5.9)
25 µM ALA	600	451 (76.8 ± 3.6)	201 (34.5 ± 1.9)	8 (4.1 ± 1.3)	77 (39.0 ± 3.5)	43 (21.4 ± 1.7)	128 (64.5 ± 3.8)
*p*-value		0.91	0.90	0.47	0.34	0.47	0.49

Values are presented as *N* (mean ± standard error). Tukey’s test was performed at a 5% probability level. Cleavage: cleavage rate (%); blastocyst: blastocyst rate (%); hatching day 7: hatching rate on day 7 (%); hatching day 8: hatching rate on day 8 (%); hatching on day 9: hatching rate on day 9 (%); total hatching: total hatching rate (%).

**Table 2:** Cleavage rates, blastocyst rates, and hatching kinetics of possible zygotes subjected to in vitro culture in the presence of different concentrations of alpha-lipoic acid (ALA).

Group	Possible Zygotes	Cleavage	Blastocyst	Hatching Day 7	Hatching Day 8	Hatching Day 9	Total Hatching
Control	443	340 (77.1 ± 2.2)	200 (46.2 ± 2.8)	17 (8.4 ± 1.6)	83 (42.3 ± 4.7)	51 (25.1 ± 3.5)	151 (75.8 ± 5.2)
2.5 µM ALA	443	326 (73.8 ± 2.6)	135 (30.9 ± 3.9)	7 (4.1 ± 1.7)	46 (35.7 ± 4.7)	35 (25.1 ± 4.1)	88 (64.8 ± 2.7)
5 µM ALA	440	339 (77.2 ± 1.9)	146 (34.2 ± 3.4)	12 (7.9 ± 2.1)	57 (39.5 ± 5.8)	29 (19.5 ± 3.2)	98 (66.9 ± 3.3)
10 µM ALA	435	334 (77.5 ± 2.6)	159 (37.3 ± 4.6)	14 (9.5 ± 3.0)	55 (34.1 ± 2.6)	43 (27.7 ± 2.2)	112 (71.4 ± 3.4)
25 µM ALA	440	320 (72.9 ± 4.9)	163 (34.3 ± 6.2)	15 (8.1 ± 2.8)	56 (34.4 ± 3.1)	50 (28.6 ± 3.7)	121 (71.0 ± 4.9)
*p*-value		0.60	0.15	0.54	0.61	0.38	0.36

Values are presented as *N* (mean ± standard error). Tukey’s test was performed at 5% probability level. Cleavage: cleavage rate (%); blastocyst: blastocyst rate (%); hatching day 7: hatching rate on day 7 (%); hatching day 8: hatching rate on day 8 (%); hatching on day 9: hatching rate on day 9 (%); total hatching: total hatching rate (%).

**Table 3:** Cleavage rates, blastocyst rates, and hatching kinetics of embryos produced in maturation and cultivation medium with or without alpha lipoic acid supplementation.

Group	COCs	Cleavage	Blastocyst	D7GI	D7G2	D8G1	D8G2	Vitrified Blastocysts
Control	1514	1163 (76.9 ± 1.0)	355 (23.8 ± 1.6)	55 (15.6 ± 1.9)	42 (11.9 ± 1.1)	24 (6.7 ± 0.6)	38 (11.4 ± 1.6)	159 (45.7 ± 2.4)
25 µM ALA	1522	1183 (77.5 ± 1.0)	359 (23.7 ± 1.0)	65 (18.3 ± 1.5)	59 (16.1 ± 2.8)	22 (6.7 ± 1.7)	31 (8.5 ± 1.3)	177 (49.6 ± 3.6)
*p*-value		0.69	0.96	0.11	0.13	0.97	0.10	0.22

Values are presented as *N* (% mean ± standard error). Tukey’s test was performed at a 5% probability level. Control: group without antioxidant addition; ALA: group with 25 µM alpha-lipoic acid supplemented to the maturation and culture media; Cleavage: cleavage rate (%); blastocyst: blastocyst rate (%); D7GI: grade I blastocyst percentage on day 7; D7GII: grade II blastocyst percentage on day 7; D8GI: grade I blastocyst percentage on day 8; D8GII: grade II blastocyst percentage on day 8; vitrified blastocysts: percentage of vitrified grade I and II blastocysts.

**Table 4:** Expansion and hatching kinetics after warming of embryos on day 7 produced in media with or without alpha-lipoic acid supplementation.

		Expansion				Hatching				
		0 h	12 h	24 h	Total	12 h	24 h	48 h	72 h	Total
D7GI	Control (*N* = 54)	0	47 (89.5 ± 4.6)	4 (7.0 ± 4.0)	51 (96.5 ± 2.4)	8 (12.2 ± 5.4)b	14 (30.6 ± 8.8)	20 (33.5 ± 8.1)	2 (4.0 ± 2.9)	44 (80.3 ± 4.4)
	ALA (*N* = 63)	2 (2.7 ± 1.9)	55 (84.4 ± 6.9)	5 (11.0 ± 5.6)	62 (98.2 ± 1.8)	27 (37.3 ± 10.4)a	14 (24.7 ± 6.6)	10 (17.1 ± 6.1)	6 (9.8 ± 3.2)	57 (88.9 ± 3.2)
*p*-value		0.182	0.549	0.571	0.604	0.047	0.600	0.127	0.201	0.131
D7GII	Control (*N* = 40)	0	33 (87.6 ± 7.9)	3 (5.4 ± 3.6)	36 (93.0 ± 5.0)	5 (10.2 ± 7.6)	11 (32.2 ± 8.3)	12 (30.3 ± 8.1)	4 (11.6 ± 6.6)	32 (84.3 ± 5.5)
	ALA (*N* = 56)	0	49 (85.0 ± 8.1)	1 (2.2 ± 2.2)	50 (87.2 ± 6.7)	10 (19.4 ± 8.6)	11 (22.9 ± 7.5)	14 (20.9 ± 6.7)	0 (0.0 ± 0.0)	35 (63.3 ± 11.6)
*p*-value			0.820	0.467	0.499	0.430	0.421	0.383	0.096	0.122

Values are presented as *N* (% mean ± standard error). The letters (a, b) indicate significant differences between treatments in D7GI embryos (*p* < 0.05). Control: group without antioxidant addition; ALA: group with 25 µM alpha-lipoic acid supplemented to the maturation and culture media; D7GI: grade I blastocysts on day 7; D7GII: grade II blastocysts on day 7.

**Table 5:** Expansion and hatching kinetics after warming of embryos on day 8 produced in media with or without alpha-lipoic acid supplementation.

		Expansion				Hatching				
		0 h	12 h	24 h	Total	12 h	24 h	48 h	72 h	Total
D8GI	Control (*N* = 24)	0	21 (90.7 ± 6.3)	1 (3.7 ± 3.7)	22 (94.4 ± 5.6)	4 (21.3 ± 11.0)	2 (11.1 ± 11.1)	6 (26.9 ± 11.1)	2 (8.3 ± 5.9)	14 (67.6 ± 12.5)
	ALA (*N* = 22)	0	16 (75.9 ± 7.9)	4 (16.7 ± 7.4)	20 (92.6 ± 5.63)	6 (29.6 ± 12.0)	8 (35.2 ± 10.9)	2 (7.4 ± 5.6)	1 (3.7 ± 3.7)	17 (75.9 ± 11.8)
*p*-value			0.162	0.135	0.818	0.617	0.142	0.138	0.515	0.634
D8GII	Control (*N* = 35)	0	31 (89.6 ± 4.3)	3 (6.7 ± 3.3)	34 (96.3 ± 3.7)	2 (5.3 ± 3.8)b	4 (10.9 ± 6.1)	8 (22.5 ± 7.3)	2 (14.8 ± 11.3)	16 (53.6 ± 10.6)
	ALA (*N* = 31)	0	30 (94.4 ± 5.6)	1 (5.6 ± 5.6)	31 (100.0 ± 0.0)	8 (24.3 ± 8.0)a	8 (32.1 ± 12.7)	5 (16.1 ± 7.2)	4 (9.9 ± 5.8)	25 (82.5 ± 11.1)
*p*-value			0.504	0.866	0.332	0.048	0.150	0.541	0.705	0.077

Values are presented as *N* (% mean ± standard error). The letters (a, b) indicate significant differences between treatments in D8GII embryos (*p* < 0.05). Control: group without antioxidant addition; ALA: group with 25 µM alpha-lipoic acid supplemented to the maturation and culture media; D8GI: grade I blastocysts on day 8; D8GII: grade II blastocysts day 8.

## Data Availability

Data are included within the article.
